# The spectrum of Epstein-Barr virus infections of the central nervous system after organ transplantation

**DOI:** 10.1186/s12985-021-01629-6

**Published:** 2021-08-06

**Authors:** Mohamed Ridha, Dylan G. Jones, David P. Lerner, Michal Vytopil, Barbara Voetsch, Joseph D. Burns, Anil Ramineni, Pooja Raibagkar

**Affiliations:** 1 Department of Neurology, University of Cincinnati, 260 Stetson, Suite 2300, Cincinnati, OH 45267 USA; 2 Division of Neurology, Lahey Hospital and Medical Center, 41 Mall Road, Burlington, MA 01805 USA; 3 Department of Neurology, Tufts University School of Medicine, 800 Washington Street, Boston, MA 02111 USA; 4 Concord Hospital Neurology Associates, 246 Pleasant Street, Concord, NH 03301 USA

**Keywords:** Epstein-Barr virus, Post-transplant lymphoproliferative disorder, Central nervous system

## Abstract

**Background:**

Epstein-Barr virus (EBV)-related neurologic complications have a diverse presentation in transplant recipients, creating diagnostic and therapeutic challenges for clinicians. In this case series, we report unique manifestations of EBV related neurologic complications following solid organ transplant and highlight pitfalls in management.

**Case presentations:**

A retrospective search of the electronic medical record of all patients from January 2015 to December 2020 who underwent solid organ transplantation and had central nervous system complications as determined by ICD-10 codes were included. Three patients with unique manifestation of EBV-related neurologic complications after liver transplantation were identified. The first was a 52-year-old man with a live-donor liver transplant 11 years prior for Budd-Chiari syndrome presented with several weeks of headache and several lesions on brain MRI; he was diagnosed with primary central nervous system post-transplant lymphoproliferative disorder. The second patient was a 63-year-old man with a deceased-donor liver transplant 16 years prior for alpha-1-antitrypsin deficiency and was found to have a stroke; he was diagnosed with EBV encephalitis. The final patient was a 75-year-old woman with a deceased-donor liver transplant six years prior for primary biliary cirrhosis who presented with four months of gait instability; she was diagnosed with EBV myelitis. A review of the literature was performed to supplement description of the different diseases.

**Conclusions:**

EBV-related central nervous infection in post-transplant patients can manifest in a variety of neurologic syndromes, which can be challenging to diagnose. Careful correlation of clinical, pathologic, and radiologic findings and a high index of suspicion are crucial in identification and appropriate management.

## Background

With the advent of organ transplantation and modern immunosuppressive therapy, clinicians frequently encounter unique manifestations of infectious diseases, owing to the interplay of the infectious agent and the altered immune response. Of these manifestations, neurologic presentations may arise inconspicuously but can be devastating.

Epstein-Barr virus (EBV) is capable of causing a diverse range of neurologic maladies [1, 2]. Cell-mediated immunity is the primary defense against EBV-infected B-cells. However, due to immunosuppressive regimens, organ transplant recipients are at increased risk. Treatment of these EBV-related complications, including those involving the central nervous system (CNS), presents a diagnostic challenge due to their protean manifestations as well as a therapeutic dilemma, balancing the risks of organ rejection with the need for immune reconstitution. The increase in prevalence of organ transplantation and development of potential future therapies in EBV post-transplant lymphoproliferative disorders including prophylactic chemo- or antiviral drugs and cytotoxic T-lymphocyte therapy necessitate clinicians to be aware of these disorders when caring for this high-risk population [3].

The aim of this case-series is to illustrate the CNS manifestations of EBV infection after organ transplantation and review the challenges and approach to diagnosis and management.

## Methods

The study was conducted at Lahey Hospital and Medical Center (LHMC) in Burlington, Massachusetts. LHMC is an academic tertiary care center with a high-volume liver transplantation program.

We performed a single-center, retrospective review of all patients between January, 2015 and December, 2020 who underwent solid organ transplantation and had CNS complications. During the study period, there were a total of 768 solid organ transplants with 516 liver transplants and 252 kidney transplants [4]. Cases were ascertained utilizing Epic SlicerDicer search of all patients admitted during this time period, including only those with international classification of disease (ICD)-10 codes consistent with organ transplantation and CNS or EBV infection (supporting information). All cases and images were reviewed and verified by two neurologists (MR and PR) to confirm accurate case adjudication. The Institutional Review Board approved this study and a waiver of informed consent was granted.

## Case presentations

### Case 1: Primary central nervous system post-transplant lymphoproliferative disorder

A 52-year-old man with a live-donor liver transplant 11 years prior for Budd-Chiari syndrome presented with several weeks of headache and diplopia. Immunosuppression consisted of tacrolimus (Table 1). Neurologic exam identified right abducens and right facial nerve palsies.

**Table 1:** Clinical characteristics of cases with central nervous system EBV infection

Patient (age/sex)	Liver allograft type	Years from transplant	Presenting symptoms (EBV syndrome)	Immunosuppression regimen
52/M	Live-donor	11	Relapsing headache, nausea/vomiting, double vision, facial numbness (PTLD)	Tacrolimus
63/M	Dead-donor	16	Encephalopathy, fall, hyperglycemia (encephalitis)	Mycophenolate mofetil, rapamycin
75/F	Dead-donor	6	Paraparesis, numbness, urinary dysfunction, falls (myelitis)	Tacrolimus, mycophenolate mofetil

EBV, Epstein-Barr virus; F, female; M, male

**Fig. 1:**
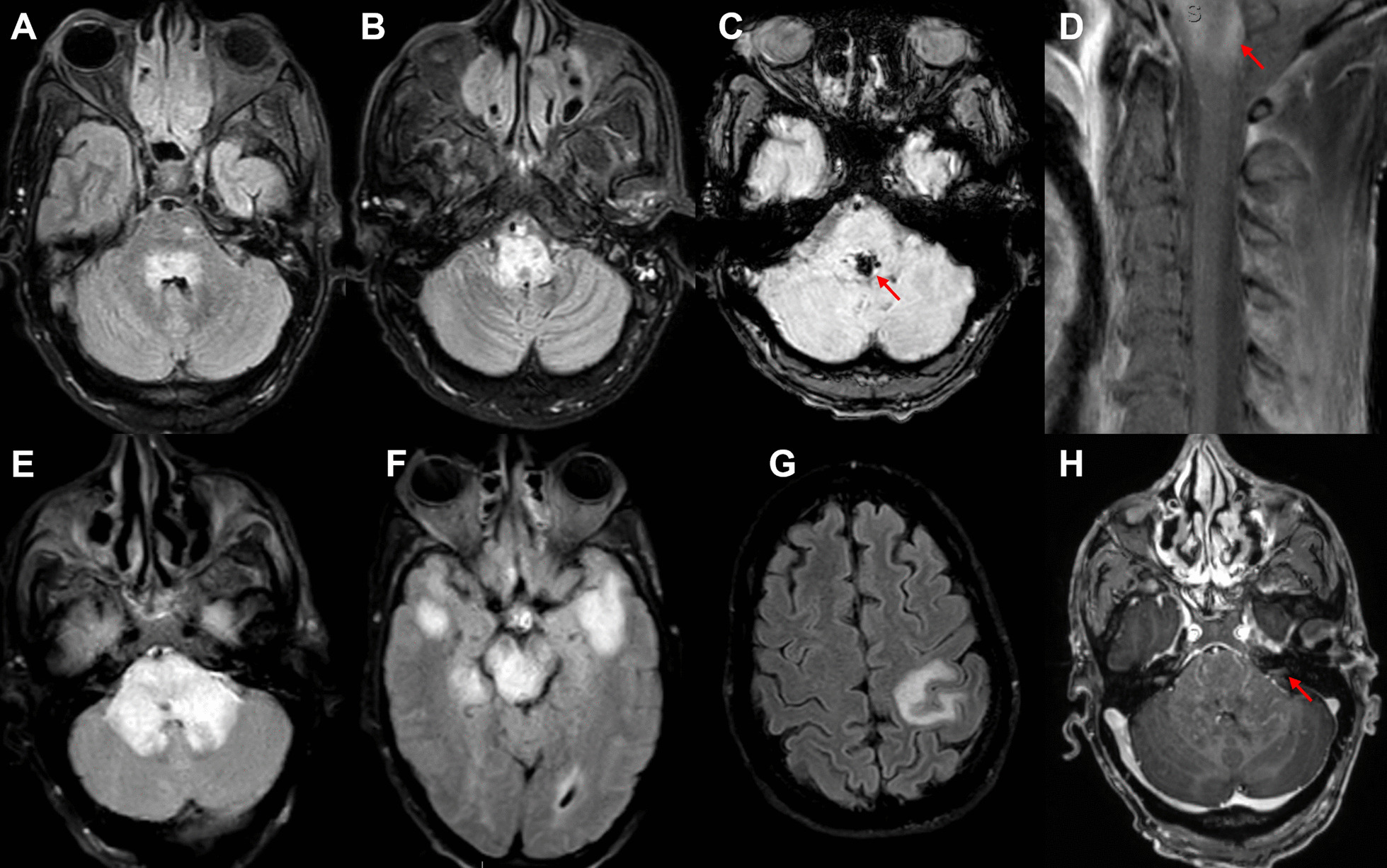
Primary CNS post-transplant lymphoproliferative disorder radiography. Initial brain MRI showing fluid attenuation inversion recovery (FLAIR) hyperintensities within the dorsal pons (**A**) and throughout the medulla (**B**) with foci of susceptibility within the pons (**C**). MRI of the cervical spine with T1 fat saturation sequence showing contrast enhancement of the upper cervical cord (**D**, arrow) with leptomeningeal enhancement. MRI one month later when the patient presented with left hemisensory symptoms showing multiple FLAIR hyperintense lesions in the pons (**E**), midbrain and bilateral temporal lobes (**F**), and left parietal lobe (**G**). Additionally, contrast enhancement within the middle cerebellar peduncles was seen (**H**)

Magnetic resonance imaging (MRI) of the brain and spinal cord revealed T2 hyperintensity involving portions of the cervical cord, brainstem, and left centrum-semiovale with leptomeningeal and right facial nerve enhancement (Fig. 1).

A thorough investigation for infectious, inflammatory, and neoplastic etiologies was performed (Table 2). EBV polymerase chain reaction (PCR) of the cerebral spinal fluid (CSF) was positive. EBV serology was positive for viral capsid antigen (VCA) IgG and early antigen (EA) IgG, but VCA IgM and Epstein-Barr nuclear antigen (EBNA) were negative, indicative of either late primary infection or prior infection.

**Table 2:** Summary of testing and outcomes of cases

Diagnosis	Case 1: post-transplant lymphoproliferative disorder (PTLD)	Case 2: EBV encephalitis	Case 3: EBV myelitis
EBV serology	EBNA IgG − EBV VCA IgM − EBV VCA IgG + EBV EA IgG +	Not performed	EBNA IgG + EBV VCA IgM ± ^b^ EBV VCA IgG + EBV EA IgG +
EBV CSF PCR	Positive	Positive (quantitative EBV PCR: 383,000 copies/mL)	Positive
CSF profile^a^	WBC 12; lymphocytes 81% (occasional plasmacytoid and atypical cells) < 1 RBC Protein 36 Glucose 53	WBC 171; lymphocytes 95% RBC 234 Protein 169 Glucose 63	WBC 23; lymphocytes 95% < 1 RBC Protein 60 Glucose 55
Other serum testing	CBC, CMP California virus, East Equine encephalitis, St. Louis encephalitis, West Equine encephalitis Treponemal antibody ANA HIV QuantiFERON Anaplasma, Ehrlichia Lyme Aspergillus galactomannan Anti-aquaporin-4 IgG Anti-MOG IgG C-reactive protein Vitamin B12	CBC, CMP Treponemal antibody HIV QuantiFERON Lyme	CBC, CMP Vitamin B12 Methylmalonic acid Copper Vitamin E Zinc HIV Lyme Treponemal antibody ANA ACE ANCA Anti-aquaporin-4 IgG Anti-MOG IgG
Other CSF testing	Herpes simplex virus I/II PCR Listeria Cryptococcal antigen Varicella zoster PCR Toxoplasma PCR Tropherma whipple Mycoplasma Histoplasma Cytomegalovirus PCR Enterovirus PCR JC virus PCR HHV-6 PCR Oligoclonal bands Cytology Flow cytometry	Herpes simplex virus I/II PCR Cryptococcal antigen Varicella zoster PCR Cytomegalovirus PCR Cytology Flow cytometry Universal bacterial and fungal PCR	Herpes simplex virus I/II PCR Cryptococcal antigen Varicella zoster PCR Cytomegalovirus PCR paraneoplastic panel VDRL oligoclonal bands West-Nile virus PCR HHV-6 PCR
Treatment	1. rituximab, prednisone, methotrexate 2. IV ganciclovir then po valganciclovir; tacrolimus held	Mycophenolate mofetil held, tacrolimus continued; IV acyclovir then transitioned to IV ganciclovir	Mycophenolate held, tacrolimus continued; IV solumedrol
Outcome	Significant improvement 20 weeks after presentation with residual radiographic lesions	Deceased	Complete recovery 5 months later

ACE, angiotensin converting enzyme; ANCA, antineutrophil cytoplasmic antibodies; ANA, antinuclear antibody; CBC, complete blood cell count; CMP, complete metabolic panel; CSF, cerebral spinal fluid; EA, early antigen; EBER, Epstein-Barr encoding region; EBNA, Epstein-Barr nuclear antigen; EBV, Epstein-Barr virus; HHV-6, Human Herpesvirus 6; HIV, human immunodeficiency virus; IV, intravenous; JC virus, John Cunningham virus; LMP1, latent membrane protein 1; MOG, myelin oligodendrocyte glycoprotein; RBC, red blood cell count; VCA, viral capsid antigen; VDRL, venereal disease research laboratory; WBC, white blood cell count

^a^Lab reference values: WBC < 10 cells/μL, RBC < 1 cells/μL, lymphocytes 40–80%, protein 15–45 mg/dL, glucose 40–70 mg/dL)

^b^+/−Means borderline/equivocal

**Fig. 2:**
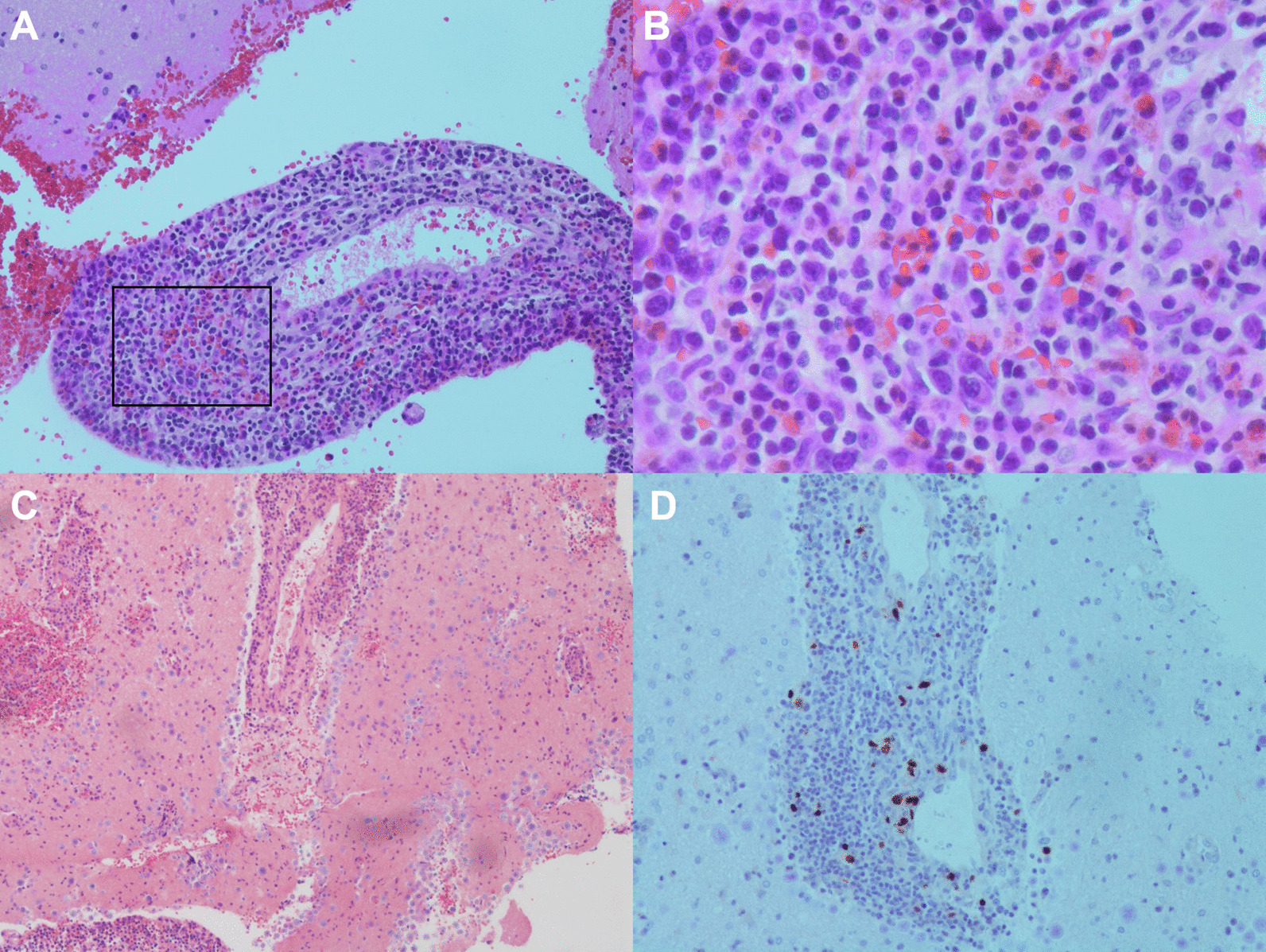
Primary CNS post-transplant lymphoproliferative disorder pathology. Pathology showed perivascular polymorphic inflammatory infiltrate, as well as numerous plasma cells and eosinophils (**A**–**C**). Panel B is an enhanced image of the boxed area in **A**. Immunohistochemical staining was positive for scattered EBER positive cells (**D**). Also positive for CD20 positive B-cells, CD3 positive T cells, polytypic plasma cells (not pictured)

Pathology from stereotactic biopsy of a left temporal lobe lesion showed perivascular polymorphic inflammatory infiltrate, including B- and T-cells. Immunohistochemical stains revealed CD20 positive B-cells, CD3 positive T cells, CD68 positive microglial nodules, plasma cells, and scattered EBV-encoded small RNAs (EBER) positive cells (Fig. 2). Latent membrane protein 1 (LMP1) was negative. These results were initially interpreted as Primary CNS Post-transplant Lymphoproliferative Disorder (PCNS-PTLD). Tacrolimus was withheld, and the patient was treated with rituximab, prednisone, and methotrexate. A subsequent opinion of the pathology changed the diagnosis in favor of EBV encephalitis, prompting initiation of ganciclovir and withholding chemotherapy. The neurologic exam and repeat imaging improved over the course of this hospitalization; tacrolimus was resumed. He was readmitted a month later with new left hemisensory loss. Brain MRI demonstrated new expansile T2 hyperintense lesions (Fig. 1). Tacrolimus was again discontinued, and valganciclovir was initiated.

After 2 weeks, he developed leukopenia (white blood cell count of 1.71 K/μL (reference 4.00–11.00 K/μL)) with severe neutropenia (absolute neutrophilic count 0.13 K/μL (reference 1.50–7.70 K/μL)) attributed to antiviral therapy. MRI brain again revealed progressive lesion enlargement. Given the longitudinal clinical, radiographic, and pathologic course, the diagnosis was revised to PCNS-PTLD. All immunosuppression and antiviral therapy were held. SubsequentMRI after one month showed regression of the lesions. At follow-up 6 months later, he remained clinically stable without relapse.

### Case 2: EBV encephalitis

A 63-year-old man with a deceased-donor liver transplant 16 years prior for alpha-1-antitrypsin deficiency presented to the emergency department after a fall.

**Fig. 3:**
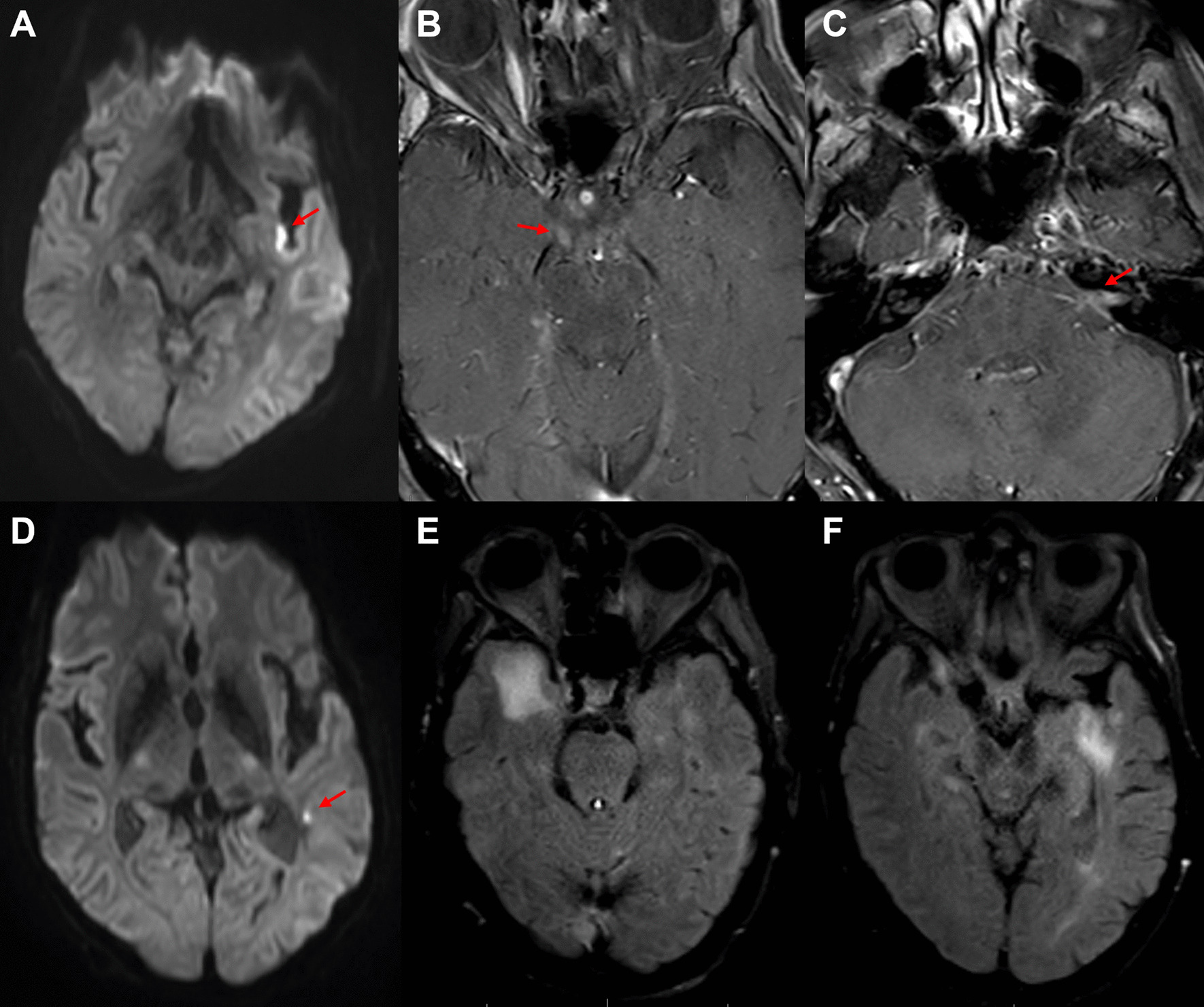
EBV encephalitis. MRI of the brain revealed a small area of diffusion restriction along the posterior left insula, consistent with an acute infarct (**A**); multiple cranial enhancement including the right oculomotor nerve (**B**, arrow), left facial and vestibulocochlear nerve complex (**C**, arrow), and left trigeminal nerve (not pictured). There were also new areas of restricted diffusion in the left frontal operculum and posterior left temporal lobe white matter (**D**, arrow). Two weeks later, MRI of the brain showed multiple areas of non-enhancing FLAIR hyperintensity in the bilateral temporal lobes (**E**, **F**)

Immunosuppression consisted of mycophenolate mofetil and sirolimus (Table 1). Physical examination identified right hemisensory loss. Brain MRI revealed an acute left insular infarct (Fig. 3). Over the initial three days of his hospitalization, he developed bilateral abducens, left facial, and right oculomotor cranial nerve palsies. Repeat MRI with contrast demonstrated new cortical infarcts and enhancement of the left facial, left trigeminal, and right oculomotor nerves (Fig. 3). Investigations (Table 2) were notable for a positive CSF EBV PCR with 383,000 copies/mL.

Mycophenolate mofetil was discontinued, sirolimus was continued, and ganciclovir was initiated. The patient became progressively obtunded over several days. MRI one week later indicated disease progression with new infarcts (Fig. 3). The patient was transitioned to hospice and died.

### Case 3: EBV myelitis

A 75-year-old woman with a deceased-donor liver transplant 6 years prior for primary biliary cirrhosis presented with four months of progressive weakness and gait instability.

Immunosuppression consisted of tacrolimus and mycophenolate mofetil (Table 1). On examination, she had paraparesis, diminished proprioception, brisk reflexes in all limbs, and bilateral Babinski sign.

**Fig. 4:**
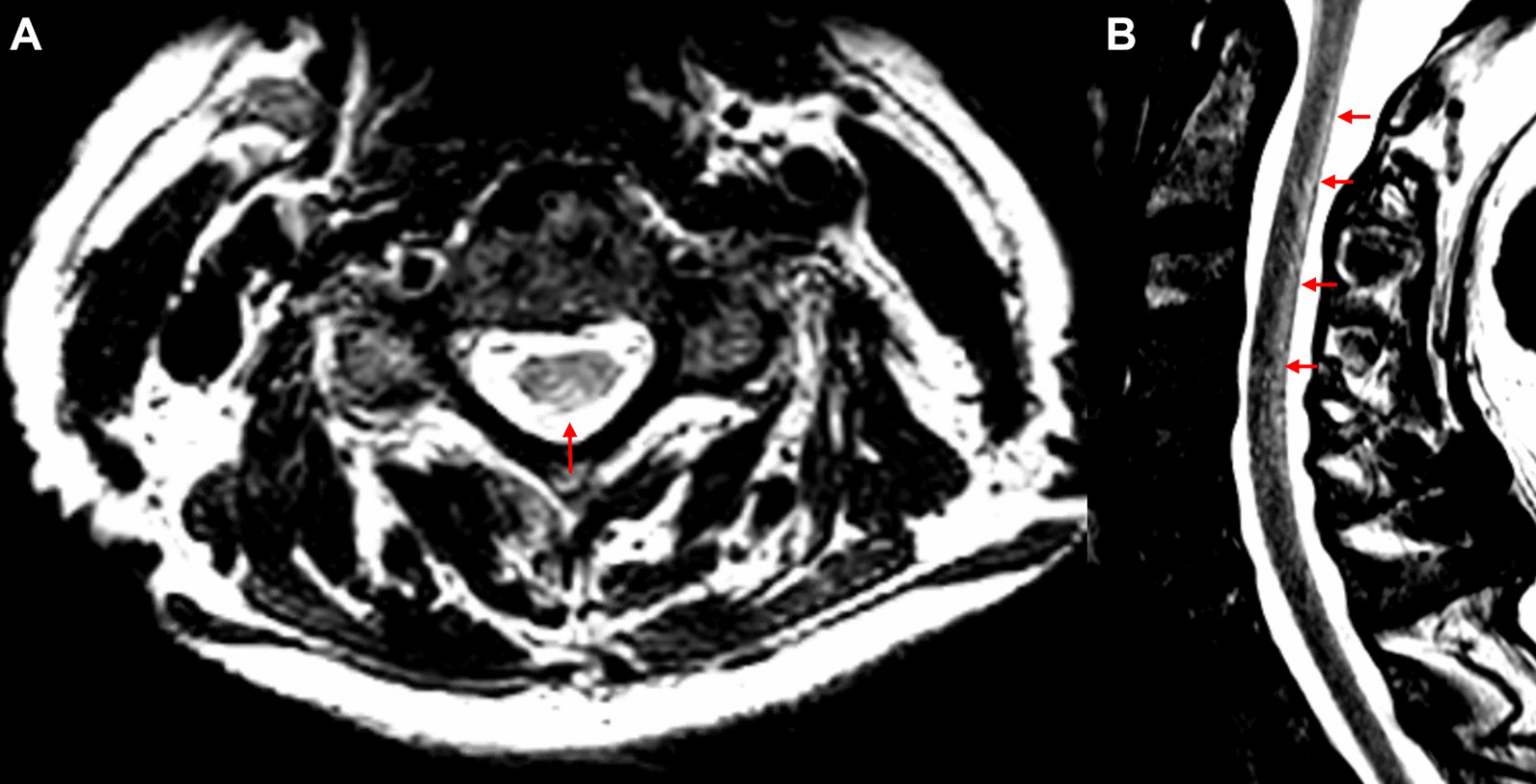
EBV myelitis. MRI of the spinal cord in axial (**A**) and sagittal (**B**) views revealed a longitudinal extensive area of T2 hyperintensity involving the dorsal cervical spinal cord from C1–C7 (arrows)

Spinal MRI demonstrated a longitudinal extensive T2 hyperintensity of the dorsal columns (C1–C7) with enhancement (Fig. 4). Investigations (Table 2) were remarkable for a positive CSF EBV PCR. EBV serology detected elevated VCA IgG, EBNA IgG, and EA IgG with borderline VCA IgM., indicating a chronic infection or viral reactivation.

She was treated with methylprednisolone. Mycophenolate mofetil was discontinued with continuation of tacrolimus. At five-month follow-up, she had mild residual paraparesis but was able to ambulate with support. Repeat MRI revealed resolution of the lesion.

## Discussion

### Virology

EBV is a DNA virus of the herpesvirus family with a tropism for B-lymphocytes. The life cycle consists of two forms—the latent and lytic phases. The latent phase results in activation, proliferation, and somatic hypermutation of infected B-cells. The phase is characterized by viral expression of proteins EBER and LMP-1. LMP-1 binds to the CD40 receptor on B-cells and prevents cellular apoptosis. These immortalized B-cells establish a viral cache. In the lytic phase, the virus replicates, destroys host cells, and infects other B-cells [1]. Remission is achieved by the control of latent-infected B-cells, primarily by the response of cytotoxic T-cells. Compromise of T-cell mediated immunity results in the loss of the ability to control dormant, immortalized, infected B-cells, causing a neoplastic proliferation in PTLD. This is analogous to pathogenesis of primary CNS lymphoma in AIDS [1].

In contrast to PTLD, EBV encephalitis and myelitis are manifestations of the lytic phase of the viral cycle. It is hypothesized that the neuronal damage is due to the infiltrative process of infected B-cells and the reactive immunologic cascade rather than a primary infection of neural cells [1].

### PCNS-PTLD

#### Epidemiology and risk factors

PTLD was initially described in 1970 and is defined by the World Health Organization as lymphoid proliferation or lymphoma occurring in patients who have undergone organ transplantation and are maintained on immunosuppressive regimens [5]. PTLD is the second most common malignancy in the post-transplant population, with approximately 15% of cases involving CNS [5, 6]. Risk factors include type of organ transplanted, greater degree of immunosuppression, EBV status of host and donor, and younger host age. Intestine and lung transplantations have the highest rate of PTLD (20%), whereas kidney and liver transplants have a lower rates (1–3%) [5, 7–10]. Seronegative hosts and EBV positive donors have increased risk likely due to the introduction of an EBV-infected graft into a naïve host [5].

#### Presentation

CNS involvement in PTLD is commonly accompanied by involvement of other organ systems; however, PCNS-PTLD may occur without evidence of systemic involvement. Incidence is highest at six months to one year post-transplant, but PCNS-PTLD may develop several years after the transplant (median 4.4 years) [5]. Presentations include increased intracranial pressure, headache, seizure, and focal neurologic deficits [5–8]. Intraocular spread may occur as well, and a slit-lamp exam is recommended.

#### Investigation modalities

Due to the immunosuppressed status of the patients and the non-specific MRI findings, a thorough evaluation of vascular, infectious, and other neoplastic etiologies such as cerebral abscess, posterior reversable encephalopathy syndrome (PRES), stroke, cerebral venous sinus thrombosis, demyelination, infectious encephalitis, autoimmune encephalitis, glioblastoma, and metastatic disease must be undertaken by treating physicians [11]. A summary of relevant testing is highlighted in Table 2.

Brain and spinal cord MRI with contrast is the imaging modality of choice. Radiographically, lesions are commonly multifocal with preferential involvement of periventricular structures with or without meningeal enhancement or hemorrhage. Lesions are typically contrast enhancing with variable patterns (homogenous, ring, or heterogeneous) [5, 11].

The CSF profile is non-specific with minimal pleocytosis and increased protein. Cytology may show malignant cells, and flow cytometry can sometimes detect a monoclonal population [12]. CSF testing alone is usually insufficient for diagnosis; tissue biopsy is needed in most cases.

Pathologic diagnosis requires the detection of latent viral proteins EBER and LMP-1 utilizing in-situ hybridization or immunohistochemistry. As in our case, it may be diagnostically challenging to differentiate EBV encephalitis from PCNS-PTLD. The pathogenies of CNS-PTLD is not binary and the disease itself exists as a continuum from early lesions which resemble reactive lymphoplasmacytic proliferation to the polymorphic subtype with mixed lymphoid and plasma cells to monomorphic PTLD with malignant lymphoid cells of single clonality [3, 10]. Furthermore, it is recognized that PTLD lesions demonstrate a mixed molecular pattern of latent and lytic EBV gene expression [13]. Thus, early CNS-PTLD presents a true diagnostic dilemma since these lesions have a histologic appearance that is difficult to distinguish from encephalitis and detection of latent EBV proteins may be scant as in our case.

#### Treatment and prognosis

The goal of therapy requires balancing control of the malignancy and preservation of graft function. It is typically recommended that the initial step be either reduction or complete withdrawal of immunosuppression [5, 10, 12]. Antiviral therapies that target thymidine kinase such as ganciclovir are ineffective since the enzymatic expression does not occur in the latent phase of the viral cycle. Immune reconstitution is typically insufficient and concurrent anti-neoplastic interventions are required. Rituximab is a preferred first-line agent given tolerability, limited toxicity, and high response rate. Other therapies include whole-brain radiation, methotrexate, and autologous or allogenic EBV-specific cytotoxic T-lymphocyte infusions [5, 6, 8–10, 12]. Response to first line therapy is the strongest predicotrs of survival, and with treatment median survival ranges from 26 to 47 months [5, 6, 8]. Current areas of investigation include prophylactic antiviral or anti-CD20 for high risk patients and the utilitity of adoptive immunotherapy in refractory disease [3].

### Non-PTLD manifestation of EBV in the CNS

#### Presentation

Presentation of EBV encephalitis, myelitis, and encephalomyelitis bears semblance to other viral infections of the CNS. Infection may be heralded by prodromal systemic manifestations of infectious mononucleosis or neurologic manifestation may be the initial symptomatology [1]. A diverse array of neurologic deficits have been described with EBV encephalitis [2]. Moreover, EBV encephalitis may trigger para-infectious acute disseminated encephalomyelitis or, as in our case, vasculitis resulting in cerebral infarcts [1, 2].

#### Investigation modalities

It is imperative that a detailed investigation be undertaken for other bacterial, viral, parasitic, and fungal etiologies in an immunocompromised patient after transplantation [14]. Other viral etiologies that may mimic EBV encephalitis or myelitis include Human herpesvirus 6, Herpes simplex virus, Varicella zoster virus, Cytomegalovirus, Human T-cell leukemia virus-1, West Nile Virus, Poliomyelitis, Powassan encephalitis, and Enterovirus A71. Other atypical neurologic infections that may be seen in the immunocompromised population include syphilis, toxoplamosmosis, tuberculosis, nocardia, cryptococcus, lyme disease, invasive fungal infections, listeriosis, whipple’s disease, and mycoplasma. Nutritional causes include vitamin B12, vitamin E, and copper deficiency. Vascular etiologies such as stroke or PRES may occur after transplant. All longitudinally extensive myelitis should entertain neuromyelitis optica, sarcoidosis, and anti-MOG encephalomyelitis in the differential. Acute disseminated encephalomyelitis may be a seen as a para-infectious, immune-mediated phenomenon after a primary EBV infection [15, 16]. Table 2 highlights relevant investigational testing.

CSF studies demonstrate a variable degree of lymphocytic pleocytosis with increased protein and normal glucose. While PCR detection of the virus is utilized for diagnosis of EBV encephalitis and myelitis a positive EBV PCR may reflect incidental reactivation [17]. Conversely, there may be false negative CSF EBV PCR [18]. Corresponding viral serologies to the viral capsid (IgG and IgM), EBNA, and EA may be helpful in supporting the presence of an acute or subacute EBV infection.

Brain MRI with contrast in EBV encephalitis may range from completely normal to multifocal areas of T2 hyperintensity in the parenchyma, diffusion restriction, or contrast-enhancement of the meninges and cranial nerves [1, 2, 19, 20]. In organ transplant recipients, EBV encephalitis has been reported to present as a tumor-like lesion [19].

For EBV myelitis, a complete spine MRI with contrast is the imaging modality of choice. There is no known pathognomonic finding, although there are reports of longitudinally extensive lesions with or without contrast enhancement [18].

The histologic findings in EBV encephalitis consist of predominantly perivascular lymphocytic infiltrates of microglia, macrophages, T-cells, and infected B-cells. The infected B-cells may show some clonality, lymphoblastoid-appearance, and increased mitotic figures, making the distinction between malignancy and infection difficult [1, 21].

#### Treatment and prognosis

The most crucial step is reduction of immunosuppression to restore the T-cell mediated immune response. There is limited evidence for the use of antiviral agents; however, their use should be strongly considered, especially in severe presentations. Intravenous ganciclovir or parenteral valganciclovir are preferred given their known activity against EBV replication in-vitro [2, 22, 23]. In organ transplant patients, valganciclovir and immunoglobulin therapy have been reported to be effective in EBV encephalitis, but valganciclovir may cause myelosuppression with prolonged use [23]. The optimal duration of treatment is unknown and is usually made based upon clinical response. Steroids are not routinely administered, and the decision should be made at the discretion of the physician.

In immunocompetent patients with EBV encephalitis, the mortality rate is approximately 10%, with most patients having good outcomes without long-term deficits [20, 24]. However, in post-transplant populations outcomes are unknown.

## Conclusions

EBV-related complications of the CNS after organ transplantation have a diverse spectrum of infectious to neoplastic manifestations. The presentation can be highly variable often leading to diagnostic delay or an incorrect initial diagnosis. Overlap between EBV encephalitis and PCNS-PTLD exists, creating an especially challenging diagnostic dilemma. A multidisciplinary approach and individualized treatment plan are fundamental for successful treatment.

## Data Availability

Data sharing is not applicable to this article as no datasets were generated or analysed during the current study.

## Abbreviations

ACE: Angiotensin converting enzyme
ANCA: Antineutrophil cytoplasmic antibodies
ANA: Antinuclear antibody
CBC: Complete blood cell count
CMP: Complete metabolic panel
CSF: Cerebral spinal fluid
EA: Early antigen
EBER: Epstein-Barr encoding region
EBNA: Epstein-Barr nuclear antigen
EBV: Epstein-Barr virus
HHV-6: Human herpesvirus 6
HIV: Human immunodeficiency virus
IV: Intravenous
JC virus: John Cunningham virus
LMP1: Latent membrane protein 1
MOG: Myelin oligodendrocyte glycoprotein
PCNS-PTLD: Primary central nervous system post-transplant lymphoproliferative
RBC: Red blood cell count
VCA: Viral capsid antigen
VDRL: Venereal disease research laboratory
WBC: White blood cell count

## References

[1] Tselis AC (2014). Epstein-Barr virus infections of the nervous system. Handb Clin Neurol.

[2] Fujimoto H, Asaoka K, Imaizumi T, Ayabe M, Shoji H, Kaji M (2003). Epstein-Barr virus infections of the central nervous system. Intern Med Tokyo Jpn.

[3] Allen UD, Preiksaitis JK (2019). Post-transplant lymphoproliferative disorders, Epstein-Barr virus infection, and disease in solid organ transplantation: guidelines from the American Society of Transplantation Infectious Diseases Community of Practice. Clin Transplant.

[4] Center - OPTN. Organ Procurement and Transplantation Network. Accessed July 5, 2021. https://optn.transplant.hrsa.gov/data/view-data-reports/center-data/#.

[5] Cavaliere R, Petroni G, Lopes MB, Schiff D, The International Primary Central Nervous System Lymphoma Collaborative Group. Primary central nervous system post-transplantation lymphoproliferative disorder: an international primary central nervous system lymphoma collaborative group report. *Cancer*. 2010;116(4):863–870. 10.1002/cncr.24834. 10.1002/cncr.24834 PMC4113953 20052713

[6] Lake W, Chang JE, Kennedy T, Morgan A, Salamat S, Başkaya MK. A case series of primary central nervous system posttransplantation lymphoproliferative disorder: imaging and clinical characteristics. *Neurosurgery*. 2013;72(6):960–970; discussion 970. 10.1227/NEU.0b013e31828cf619. 10.1227/NEU.0b013e31828cf619 PMC4442016 23685504

[7] Castellano-Sanchez AA, Li S, Qian J, Lagoo A, Weir E, Brat DJ (2004). Primary central nervous system posttransplant lymphoproliferative disorders. Am J Clin Pathol.

[8] Evens AM, Choquet S, Kroll-Desrosiers AR (2013). Primary CNS posttransplant lymphoproliferative disease (PTLD): an international report of 84 cases in the modern era. Am J Transplant Off J Am Soc Transplant Am Soc Transpl Surg.

[9] Petrara MR, Giunco S, Serraino D, Dolcetti R, De Rossi A (2015). Post-transplant lymphoproliferative disorders: from epidemiology to pathogenesis-driven treatment. Cancer Lett.

[10] Heslop HE (2009). How I treat EBV lymphoproliferation. Blood.

[11] White ML, Moore DW, Zhang Y, Mark KD, Greiner TC, Bierman PJ (2019). Primary central nervous system post-transplant lymphoproliferative disorders: the spectrum of imaging appearances and differential. Insights Imaging.

[12] Kittan NA, Beier F, Kurz K (2011). Isolated cerebral manifestation of Epstein-Barr virus-associated post-transplant lymphoproliferative disorder after allogeneic hematopoietic stem cell transplantation: a case of clinical and diagnostic challenges. Transpl Infect Dis Off J Transplant Soc.

[13] Montone KT, Hodinka RL, Salhany KE, Lavi E, Rostami A, Tomaszewski JE (1996). Identification of Epstein-Barr virus lytic activity in post-transplantation lymphoproliferative disease. Mod Pathol Off J US Can Acad Pathol Inc.

[14] Pruitt A (2018). Central nervous system infections complicating immunosuppression and transplantation. Contin Lifelong Learn Neurol.

[15] Bahadori HR, Williams VC, Turner RP (2007). Acute disseminated encephalomyelitis following infectious mononucleosis. J Child Neurol.

[16] Mohsen H, Abu Zeinah GF, Elsotouhy AH, Mohamed K (2013). Acute disseminated encephalomyelitis following infectious mononucleosis in a toddler. BMJ Case Rep.

[17] Weinberg A, Bloch KC, Li S, Tang Y-W, Palmer M, Tyler KL (2005). Dual Infections of the central nervous system with Epstein-Barr virus. J Infect Dis.

[18] Caldas C, Bernicker E, Nogare AD, Luby JP (1994). Case report: transverse myelitis associated with Epstein-Barr virus infection. Am J Med Sci.

[19] Khalil M, Enzinger C, Wallner-Blazek M (2008). Epstein-Barr virus encephalitis presenting with a tumor-like lesion in an immunosuppressed transplant recipient. J Neurovirol.

[20] Abul-Kasim K, Palm L, Maly P, Sundgren PC (2009). The neuroanatomic localization of Epstein-Barr virus encephalitis may be a predictive factor for its clinical outcome: a case report and review of 100 cases in 28 reports. J Child Neurol.

[21] Schellinger PD, Sommer C, Leithäuser F (1999). Epstein-Barr virus meningoencephalitis with a lymphoma-like response in an immunocompetent host. Ann Neurol.

[22] Zarlasht F, Salehi M, Abu-Hishmeh M, Khan M (2017). Encephalitis treatment - a case report with long-term follow-up of EBV PCR in cerebrospinal fluid. Int J Gen Med.

[23] Lau JSY, Low ZM, Abbott I (2017). Epstein-Barr virus encephalitis in solid organ transplantation. New Microbiol.

[24] Dyachenko P, Smiianova O, Kurhanskaya V, Oleshko A, Dyachenko A (2018). Epstein-barr virus-associated encephalitis in a case-series of more than 40 patients. Wiadomosci Lek Wars Pol 1960.

